# Multi-Party Semi-Quantum Simultaneous Ascending Auction Protocol Based on Single-Particle States

**DOI:** 10.3390/e28010039

**Published:** 2025-12-28

**Authors:** Xiuqi Wu, Yu Yang, Baichang Wang, Yue Zhang, Yunguang Han

**Affiliations:** 1 College of Computer Science and Technology, Nanjing University of Aeronautics and Astronautics, Nanjing 211106, China; 2 School of Computer Engineering, Nanjing Institute of Technology, Nanjing 211167, China

**Keywords:** quantum auction, semi-quantum protocol, quantum information security, simultaneous ascending auction, unconditional security

## Abstract

Simultaneous ascending auctions find extensive applications in spectrum licensing and advertising space allocation. However, existing quantum sealed-bid auction protocols suffer from dual limitations: they cannot support multi-item simultaneous bidding scenarios, and their reliance on complex quantum resources along with requiring full quantum operational capabilities from bidders fails to accommodate practical constraints of quantum resource-limited users. To address these challenges, this paper proposes a multi-party semi-quantum simultaneous ascending auction protocol based on single-particle states. The protocol employs a trusted honest third party (HTP) responsible for quantum state generation, distribution, and security verification. Bidders determine their groups through quantum measurements and privately encode their bid vectors. Upon successful HTP authentication, each bidder obtains a unique identity code. During the bidding phase, HTP dynamically updates quantum sequences, allowing bidders to submit bids for multiple items by performing only simple unitary operations. HTP announces the highest bid for each item in real time and iteratively generates auction sequences until no new highest bid emerges, thereby achieving simultaneous ascending auctions for multiple items. It acts as a quantum-secured signaling layer, ensuring unconditional security for bid transmission and identity verification while maintaining classical auction logic. Quantum circuit simulations validate the protocol’s feasibility with current technology while satisfying critical security requirements, including anonymity, verifiability, non-repudiation, and privacy preservation. It provides a scalable semi-quantum auction solution for resource-constrained scenarios.

## 1. Introduction

Quantum cryptography, a key subfield of quantum information science, combines quantum-mechanical principles with classical cryptography. It has achieved major advances in protocol design and implementation, including Quantum Key Distribution (QKD) [1,2], Quantum Secure Direct Communication (QSDC) [3,4], and Quantum Secret Sharing (QSS) [5,6]. These technologies are increasingly deployed in practice, such as quantum voting systems [7] and quantum transaction platforms, offering new models for secure applications.

Auctions are fundamental mechanisms for resource allocation and trading [8,9,10]. By information transparency and bidding rules, they can be categorized into open-cry and sealed-bid formats [11]. English and Dutch auctions are open-cry, with real-time price discovery. Sealed-bid auctions require bidders to submit offers independently and confidentially, which better preserves strategy privacy and suits anonymity-sensitive scenarios.

However, conventional sealed-bid mechanisms rely on computational assumptions in classical cryptography (e.g., integer factorization and discrete logarithms). With rapid progress in quantum computing [12,13], Shor’s algorithm solves these problems in polynomial time [14] and Grover’s algorithm speeds up search [15], exposing sealed-bid systems to quantum attacks [16]. This motivates security protocols grounded in physical principles.

Unlike classical schemes that offer computational security, quantum cryptographic protocols provide information-theoretic security (or unconditional security). This ensures that encrypted data remains secure even against adversaries with unlimited computing power, guaranteeing forward secrecy. In the context of auctions, this prevents historical bid data from being decrypted retroactively once powerful quantum computers become available. It is important to clarify that our proposed protocol serves as a quantum-secured signaling layer. It utilizes quantum mechanics to secure the transmission of bids and verify identities, while the underlying auction allocation logic (comparing bids and determining winners) follows the classical simultaneous ascending auction mechanism.

Against this backdrop, research on quantum sealed-bid auction (QSA) protocols has grown rapidly. The goal is to design quantum-resilient mechanisms that ensure fairness, preserve privacy, and enhance security.

The conceptual framework of quantum auctions was introduced in 2008 [17]. In 2009, Naseri [18] proposed a GHZ-based QSA enabling secure multi-party bid transmission. That scheme resisted only external attacks and was vulnerable internally. Follow-up work refined designs and security analyses. Qin et al. [19] and Yang et al. [20] exposed attacks via double CNOT and pseudo-entangled states; their fixes did not consider a dishonest auction center. In 2010, Zhao et al. [21] added post-confirmation to mitigate collusion, later refined for partial collusion. In 2014, Liu et al. [22] integrated QKD for bid encryption and hash-based confirmation. In 2016, they proposed a single-particle QSBA [23], reducing quantum resources, but Zhang et al. [24] showed coordinated attacks remained. Recent advances include QFT- and *d*-dimensional-state protocols [25], an anonymous QSA via the Chinese Remainder Theorem [26], and an auctioneer-free scheme with Bell-state swapping [27]. Semi-quantum secure multi-party summation (SQSMS) [28,29,30,31,32,33] has also gained attention due to its compatibility with users of varying quantum capabilities.

Despite these advancements, existing QSA research faces two primary “shortages”: (1) **Capability Shortage**—most protocols require fully quantum-capable participants, which is impractical for general users. (2) **Functional Shortage**: Most studies focus on single-item sealed-bid auctions, failing to address the complexity of simultaneous ascending auctions for multiple items. To address this, we combine Milgrom’s simultaneous ascending auction theory [34] with a semi-quantum design and propose a quantum simultaneous ascending auction (QSAA) framework that supports users with diverse capabilities. Under capability constraints, semi-quantum bidders obtain unique identity codes from an honest, fully quantum third party (HTP) and submit bids using simple unitary operations. The protocol preserves anonymity, privacy, fairness, verifiability, and non-repudiation: only HTP learns bids; sealed bids are disclosed simultaneously; bidders cannot repudiate submitted bids and HTP cannot deny receipts.

The remainder of this paper is organized as follows: Section 2 introduces the fundamental concepts of simultaneous ascending auction mechanisms and semi-quantum protocols. Section 3 elaborates on a multi-party semi-quantum simultaneous ascending auction protocol in detail. Section 4 presents simulation analyses. Section 5 provides performance evaluations. Finally, Section 6 concludes the paper.

## 2. Preliminaries

Our protocol enables anonymous auctions for users with limited quantum capabilities. The semi-quantum simultaneous ascending auction (SQSAA) assigns each bidder a unique identity code. Bidders apply unitary operations to encode bid prices onto quantum sequences, implementing a simultaneous ascending-price auction. We first introduce the auction mechanism and the semi-quantum model used in this paper.

### 2.1. Simultaneous Ascending Auction Mechanism

The simultaneous ascending-price auction is a multi-item dynamic mechanism widely used in spectrum licensing and advertisement allocation. Its core characteristics include simultaneous bidding for multiple items, round-by-round price increments, and a public bidding process. In each round, bidders may raise bids on any items. After each round, each item’s price updates to the highest bid received. The process repeats until no further bids are submitted, and items are allocated to the highest bidders at their final prices.

### 2.2. Semi-Quantum Protocol Architecture and Trust Model

The semi-quantum architecture involves two types of users:

**Quantum Users (HTP)**—the honest third party (HTP) possesses full quantum capabilities (preparation, manipulation, and measurement). Assumption: In this semi-quantum model, the HTP is assumed to be a trusted authority. The HTP generates and measures states correctly and does not collude with bidders to reveal bids to others, although the HTP itself learns the bid values.

**Semi-Quantum Users (Bidders):** Participants with constrained quantum capabilities, typically limited to Z-basis or X-basis measurements (computational or Fourier bases), applying simple unitary operations, and reflecting received quantum states. This design reduces hardware complexity and cost.

### 2.3. Protocol Characteristics

The semi-quantum design offers two practical advantages [30]:

System compatibility—basic quantum operations can be implemented using low-cost optical components. When devices malfunction, systems can fall back to a semi-quantum mode, enhancing robustness and easing the transition from classical to quantum infrastructures.

Cost efficiency: Limiting some users’ quantum capabilities reduces dependence on expensive quantum resources and large-scale quantum memory processing, enabling economically viable deployment during the current transitional phase.

## 3. Multi-Party Semi-Quantum Simultaneous Ascending Auction Protocol

Assume an honest third party (HTP) with full quantum capability acts as the auction coordinator and never colludes with bidders. There are *n* semi-quantum participants $P_{1},P_{2},\ldots ,P_{n}$ who serve as bidders with restricted capabilities: computational-basis or Fourier-basis measurement, simple unitary operations, and reflection. Each bidder $P_{i}\;\left(i=1,2,\ldots ,n\right)$ independently generates a private randomized identity vector $v_{i}$ satisfying $v_{i}=\left(v_{i}^{1},v_{i}^{2},\ldots ,v_{i}^{n}\right),v_{i}^{o}\in \left\{0,1\right\},o=\left(1,2,\ldots ,n\right).$ These vectors serve as cryptographic identity anchors, dynamically integrated into quantum state manipulations through controlled unitary operations.

**Figure 1:**
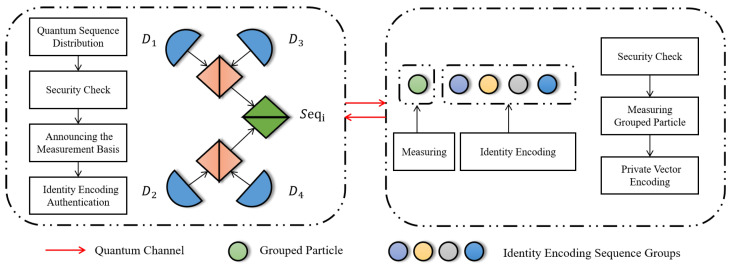
Workflow of identity encoding distribution and verification at HTP. (Lateral diagrams correspond to Step 4 and 5).

The protocol has four phases: initialization, identity encoding distribution, bidding, and result announcement. In initialization, HTP constructs differentiated quantum sequences for each bidder and collaborates with $P_{i}$ to validate channel security. In identity encoding distribution, each bidder generates a private identity vector, applies unitary operations to assigned particles, HTP constructs a verification matrix, and validates integrity against predefined rules. In bidding, each bidder applies unitary operations to encode bids and computes a commitment. In the result announcement, HTP determines the highest bid and bidder per item, and others verify using commitments. The schematic diagram of the protocol is shown in Figure 1.

### 3.1. Initialization Phase

**Step 1:** HTP and each authorized bidder pre-share an authenticated key sequence $K_{i}=\left(k_{i}^{1},k_{i}^{2},\ldots ,k_{i}^{4n+1+m}\right),\;k_{i}^{l}\in \left\{0,1\right\}$. * Note: These keys are distributed via a secure classical channel or a prior standard QKD session before the auction begins. If keys are compromised, the authentication fails; thus, key refreshment is recommended for subsequent sessions. * HTP randomly prepares (4n+1) rotation-basis particles $R_{i}=\left(r_{i}^{1},r_{i}^{2},\ldots ,r_{i}^{4n+1}\right),\;r_{i}^{p}\in \left\{|0\rangle ,|1\rangle ,|+\rangle ,|-\rangle \right\}$ and *m* decoy particles $C_{i}=\left(c_{i}^{1},\ldots ,c_{i}^{m}\right),\;c_{i}^{q}\in \left\{|0\rangle ,|1\rangle ,|+\rangle ,|-\rangle \right\}$ for each bidder. According to $K_{i}$, HTP determines the embedding order of decoy and rotation particles in the quantum sequence, using (1) if $k_{i}^{l}$ = 0, a rotational particle $r_{i}^{p}$ is inserted into the sequence. (2) If $k_{i}^{l}$ = 1, a decoy particle $c_{i}^{q}$ is embedded instead. HTP then produces a quantum sequence $Seq_{i}$ of length (4n+1+m). Repeating this for all bidders yields *n* sequences $\left(Seq_{1},\ldots ,Seq_{n}\right)$, which HTP sends over authenticated quantum channels.

**Step 2:** Upon receiving $Seq_{i}$, bidder $P_{i}$ routes each particle according to $k_{i}^{l}$: If $k_{i}^{l}=1$, $P_{i}$ directly reflects the particle to HTP; if $k_{i}^{l}=0$, $P_{i}$ retains the particle for Step 4.

**Step 3:** HTP measures the reflected particles using the preparation bases indicated by $k_{i}^{l}$ and computes the quantum bit error rate (QBER) by comparing outcomes with prepared states. If QBER exceeds a threshold η, the protocol aborts; otherwise it proceeds. Threshold Selection: The threshold η is not arbitrary; it is selected based on standard security proofs for BB84-type protocols. To tolerate environmental noise while securely detecting intercept-resend attacks (which theoretically induce ∼25% error), η is typically set strictly below the coherent attack bound (approx. 11%), for example, in the range of 5–8% in practical scenarios.

### 3.2. Identity Encoding Distribution Phase

**Step 4:** After eavesdropping detection, each $P_{i}$ discards decoy particles and keeps rotation particles. HTP designates the first rotation particle as the grouping particle and publishes its measurement basis on the bulletin board. HTP records initial states and counts bidders per group. Each $P_{i}$ measures the grouping particle according to determine its group: outcomes $|0\rangle ,|1\rangle ,|+\rangle ,|-\rangle$ map to groups 1–4.

**Step 5:** Each $P_{i}$ generates a private *n*-dimensional vector $v_{i}$, then applies unitary operations to assigned particles according to: (1) if $v_{i}^{o}$ = 0, it applies the Pauli-I operation ($\sigma_{I}$) to the rotational particle. (2) If $v_{i}^{o}$ = 1, it performs the Pauli-Y operation ($\sigma_{Y}$) on the rotational particle.

Afterward, $P_{i}$ sends the updated sequence $Seq_{i}^{\prime }$ to HTP.

**Step 6:** Upon receiving $Seq_{i}^{\prime }$ from all bidders, HTP determines each bidder’s group from $k_{i}^{l}$ and the grouping particles, then measures each particle in the corresponding basis to form a mapping $f_{x}:x\mapsto z_{i}^{j}$ (Table 1). For both computational and Fourier bases, record $z_{i}^{j}=1$ if the outcome flips relative to the initial state; otherwise, set $z_{i}^{j}=0$.

(1) $$Z=\left[\begin{array}{cccc} z_{1}^{1} & z_{1}^{2} & \ldots & z_{1}^{4n}\\ \vdots & \vdots & \ddots & \vdots \\ z_{n}^{1} & z_{n}^{2} & \ldots & z_{n}^{4n} \end{array}\right]$$

After constructing the n×4n matrix from $z_{i}^{j}$ of the quantum sequences $Seq_{i}^{\prime }$ returned by bidders, HTP checks the Hamming weight of each valid column. Identity encoding distribution succeeds iff, for every valid column *j*,

(2) $$\sum_{i=1}^{n}z_{i}^{j}=1$$

and the number of bidders within each group matches HTP’s initial records. Liveness and Restart: If this condition is not met (e.g., column sum ≠1) or if the number of bidders in a group mismatches the record (group collision), HTP broadcasts a “Restart” signal. The current round is discarded, and the Identity Encoding Phase repeats from Step 1. This ensures the protocol remains live and reaches a valid distribution state. Columns with no embedded identity encoding are excluded from verification.

### 3.3. Bidding Phase

**Step 7:** Completing these operations, each bidder obtains a unique identity code. Repeating Steps 1–3, HTP generates a new quantum sequence ${\mathrm{Seq}}_{i}^{\left(r\right)}$ and new $K_{i}^{\left(r\right)}$ for each round *r*. In each sequence, the first (2+n) particles encode identity (2 group particles followed by *n* identity particles). For item *w* with domain $\left\{0,\ldots ,d_{w}-1\right\}$, the next $\left\lceil \log_{2}d_{w}\right\rceil$ particles encode the bid.

**Step 8:** Assuming HTP specifies that the initial bid range for the *w*-th auctioned item lies within $\left[0,d_{w}-1\right]$. HTP broadcasts the valid bidding range and specifies the required bit-length $L=\lceil \log_{2}d_{w}\rceil$ for the bid vector. This ensures all bidders encode their bids using a consistent quantum sequence length. Each bidder $P_{i}$ converts their bid into a corresponding private bid binary sequence $b_{i}^{\left(w\right)}=\left(b_{i}^{\left\lceil \log_{2}d_{w}\right\rceil },\ldots ,b_{i}^{2},b_{i}^{1}\right)$.

Each bidder first encodes their identity sequence. Based on the grouping of particles, the first two particles encode the group identifier, with the conventions 00, 01, 10, and 11 designating the first, second, third and fourth groups, respectively. For example, for group 2 (01), apply *I* to the first qubit and *H* to the second. The subsequent *n* particles encode the private identity code.

For all items, bits are encoded from most significant to least significant (left to right in the vector, applied right to left on qubits as specified in Step 9).

**Step 9:** The bidder applies the Hadamard, Pauli-Y and Pauli-I operations to the first two particles and encodes their identity vector. Subsequently, they encode their private bid binary sequence $b_{i}^{a}$ (where $a=1,2,\ldots ,\lceil \log_{2}d_{w}\rceil$) onto the specific bid particle sequence from right to left. If $b_{i}^{a}$ = 0, $P_{i}$ performs the $\sigma_{I}$ operation; if $b_{i}^{a}$ = 1, $P_{i}$ applies the $\sigma_{Y}$ operation. Otherwise (if no bid is intended), the bidder performs no operations on any particles in the specific bid particle sequence. Then, $P_{i}$ returns the newly processed particle sequence ${\mathrm{Seq}}_{i}^{\left(r^{\prime }\right)}$ to HTP, particle by particle. It is noteworthy that for multiple auction items, the initial-round bidding prices are encoded in the particle sequence following the first auctioned item. Correspondingly, the particle count of ${\mathrm{Seq}}_{i}^{\left(r\right)}$ is augmented according to the same rule established for the first item.

**Step 10:** Concurrently, in each round, each bidder $P_{i}$ computes a binding, hiding commitment to their bid for each item using a collision-resistant hash function Hash(·). Let $s_{i}^{w}\in Z_{N}$ be a per-round random nonce. Define ${\mathrm{com}}_{i}^{w}=\mathrm{Hash}\;\left(\mathrm{round}\_\mathrm{id}\;∥\;w\;∥\;{\mathrm{id}}_{i}\;∥\;b_{i}^{w}\;∥\;s_{i}^{w}\right),$ where $b_{i}^{w}$ is the bidder’s decimal bid for item *w* in that round, and ∥ denotes concatenation. HTP records ${\mathrm{com}}_{i}^{w}$ for each bidder and item per round. Any bid update must include a fresh commitment.

### 3.4. Result Announcement Phase

**Step 11:** After receiving the quantum sequences ${\mathrm{Seq}}_{i}^{\left(r^{\prime }\right)}$ from all bidders, HTP first constructs a mapping result matrix according to the identity encoding distribution rules and verifies each bidder’s identity encoding sequence. Subsequently, HTP measures the first two particles using the following rules. When the Pauli-I operation is applied to a qubit, the HTP measures it in the same basis as its initial state. Conversely, when the Hadamard operation is applied, measurement occurs in the conjugate basis relative to the initial state.

Taking $P_{2}$ as an example, its identity code is Group 2, Number 3. The unitary operation for its group particles consists of applying the Pauli-I operation to the first particle and the Hadamard operation to the second particle. The HTP measures the first particle using the same measurement basis as its initial state. Regardless of the initial state, if the measurement outcome matches the initial state, the HTP calculates the mapping result of the first particle as 0. For the second particle, the HTP employs the measurement basis orthogonal to the initial state: if the initial state is |0〉 or |1〉, the measurement outcome will be |+〉 or |−〉; conversely, if the initial state is |+〉 or |−〉, the measurement outcome will be |0〉 or |1〉. In this case, the HTP calculates the mapping result of the second particle as 1, as illustrated in the diagram. Please refer to Table 2 for detailed information.

Then, HTP measures the specific bid particle sequence from right to left using the corresponding basis, recording each outcome $t_{i}^{a}$: if the measurement result is opposite to the initial state, it is recorded as $t_{i}^{a}$ = 1; otherwise, $t_{i}^{a}$ = 0. Finally, HTP arranges all specific bid results $t_{i}^{a}$, converts the bits to a decimal bid, selects the highest first-round bid, and publishes it on the bulletin board (or via a classical channel).

**Step 12:** After completing initial bidding for all auctioned items, bidders collectively negotiate the bid increment range for subsequent rounds.Specifically, suppose they determine the starting price for each new round as 10% of the previous round’s highest bid for that item, $\mathrm{Starting}\;{\mathrm{Price}}_{\mathrm{next}}=\left(1+10\%\right)\times \mathrm{Highest}\;{\mathrm{Bid}}_{\mathrm{previous}}$. Then, they repeat Steps 7–10 for the next round. Upon receiving quantum sequences, HTP executes Step 11.

If a bid does not exceed the current highest bid, HTP withholds updates. The auction concludes when no item receives a higher bid; HTP then publishes all final highest bids.

**Figure 2:**
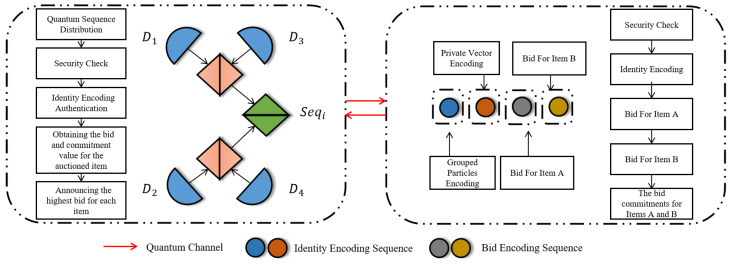
Overall simultaneous ascending auction workflow with identity and bid encoding.

**Step 13:** Assume bidder $P_{e}$ is the highest bidder for item *w*. He discloses $b_{e}^{w}$ and $s_{e}^{w}$, enabling others to recompute ${\mathrm{com}}_{e}^{w}=\mathrm{Hash}\;\left(\mathrm{round}\_\mathrm{id}\;∥\;w\;∥\;{\mathrm{id}}_{e}\;∥\;b_{e}^{w}\;∥\;s_{e}^{w}\right)$ and check it matches the recorded commitment. If $P_{e}$’s commitment is validated by all bidders and HTP, he is confirmed as the highest bidder; otherwise, verification fails. If any bidder $P_{f}$ (f≠e) claims their bid exceeds HTP’s published highest bid or $P_{e}$, $P_{f}$ broadcasts a complaint and discloses $\left(b_{f}^{w},s_{f}^{w}\right)$ for verification. Successful verification mandates HTP to update the item’s highest bid; failed verification maintains the current highest bid. HTP declares $P_{e}$ the winner for item *w* only when no valid complaints are substantiated (Figure 2).

## 4. Simulation of the Proposed Protocol

The Qiskit simulation presented here aims to validate the correctness of the quantum physical layer—specifically the single-particle encoding, decoding, and noise robustness—rather than to simulate the classical arithmetic logic of the auctioneer.

We illustrate the protocol with Qiskit simulations in two phases: multi-party semi-quantum identity encoding distribution and multi-party semi-quantum simultaneous ascending auction. Consider an HTP coordinating three semi-quantum bidders $P_{1},P_{2},P_{3}$. Bidders independently sample private vectors $v_{1}=\left(0,1,0\right),\;v_{2}=\left(0,1,1\right),\;v_{3}=\left(1,0,1\right)$.

### 4.1. Multi-Party Semi-Quantum Identity Encoding Distribution

Assume at this stage, HTP allocates distinct key sequences $K_{1},K_{2},K_{3}$ to bidders $P_{1},P_{2},P_{3}$ respectively, where

$$\begin{array}{cc} K_{1} & =\{0,1,0,1,0,1,0,0,1,0,0,1,0,1,0,0,1,0,0,0\}\\ K_{2} & =\{1,0,0,0,1,1,0,1,0,0,0,0,1,0,1,0,0,0,0,1\}\\ K_{3} & =\{1,1,0,1,0,0,0,0,1,0,1,1,0,0,0,1,0,0,0,0\} \end{array}$$

**Figure 3:**
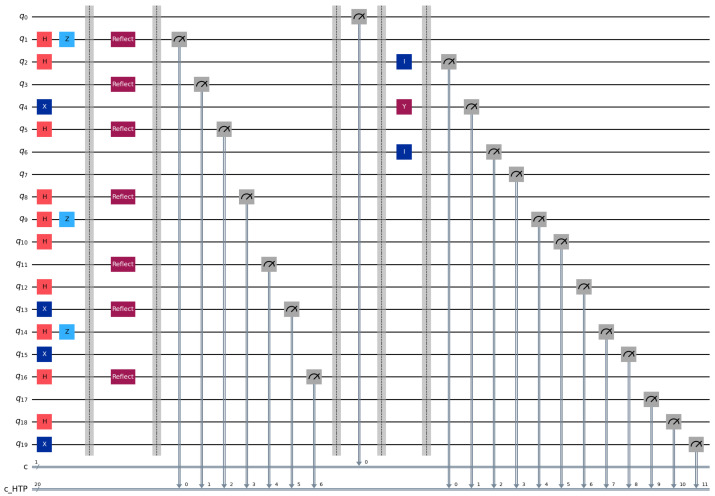
Circuit for identity encoding distribution using BB84 single-qubit states.

**Step 1:** HTP randomly generates rotation particles $R_{1},R_{2},R_{3}$ and decoy particles $C_{1},C_{2},C_{3}$ from BB84 state $\left\{|0\rangle ,|1\rangle ,|+\rangle ,|-\rangle \right\}$. According to the rules specified in the aforementioned protocol, the quantum sequences $Seq_{1}$, $Seq_{2}$ and $Seq_{3}$ are formed in Table 3. The quantum circuit diagram for $P_{1}$ is illustrated in Figure 3 according to the aforementioned conditions. In this simulation, we employ a decoy state ratio of approximately 50% (1:1 with rotation particles) to maximize statistical detection sensitivity for the case study.

**Figure 4:**
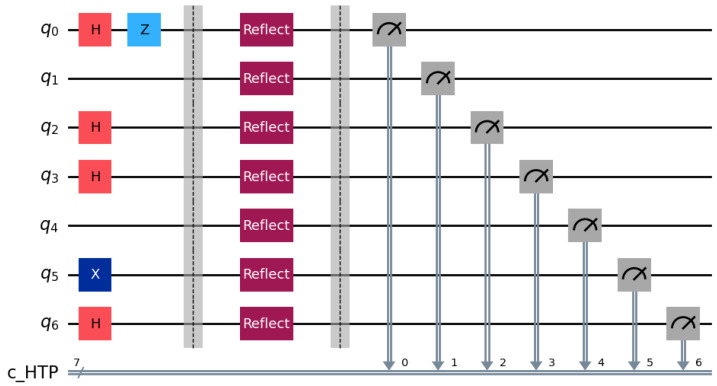
Decoy particle reflection and QBER estimation circuit used for channel checking.

**Figure 5:**
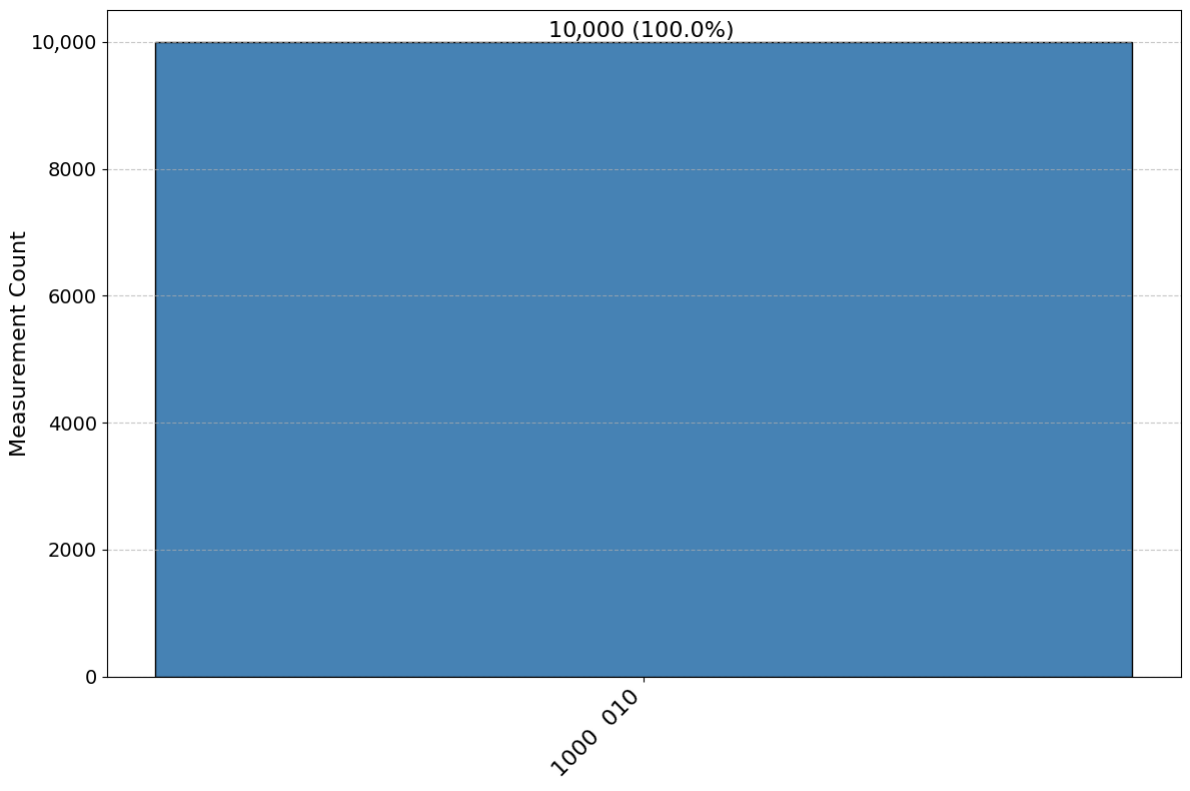
Example of positions reflected by bidder $P_{1}$ for decoy checking according to $K_{1}$.

**Step 2:** Each bidder routes particles according to $K_{i}$. $P_{i}$ reflects decoy particles and retains rotation particles. HTP measures reflected particles in the corresponding bases and computes QBER. For example, in $Seq_{1}$ the particles at positions 2, 4, 6, 9, 12, 14, and 17 are reflected (Figure 4). If the error rate is below the threshold, the protocol proceeds (Figure 5).

**Figure 6:**
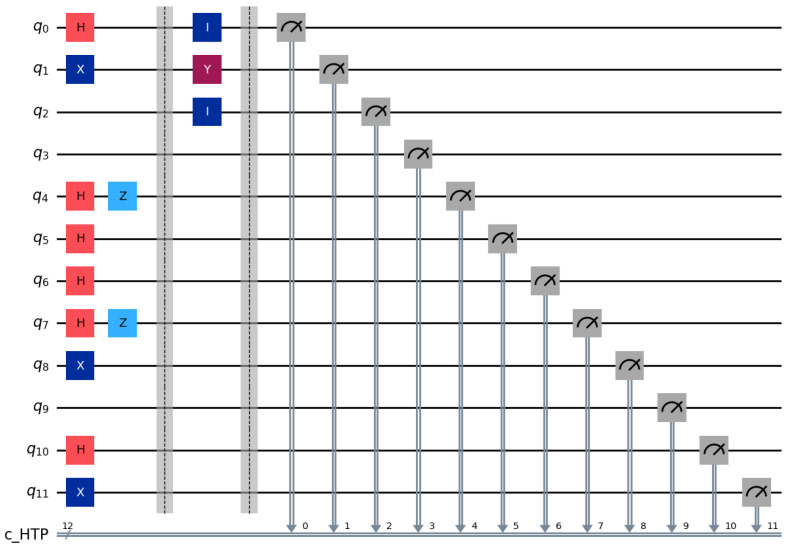
Circuit example of rotation-basis particle operations for identity and bid encoding.

**Figure 7:**
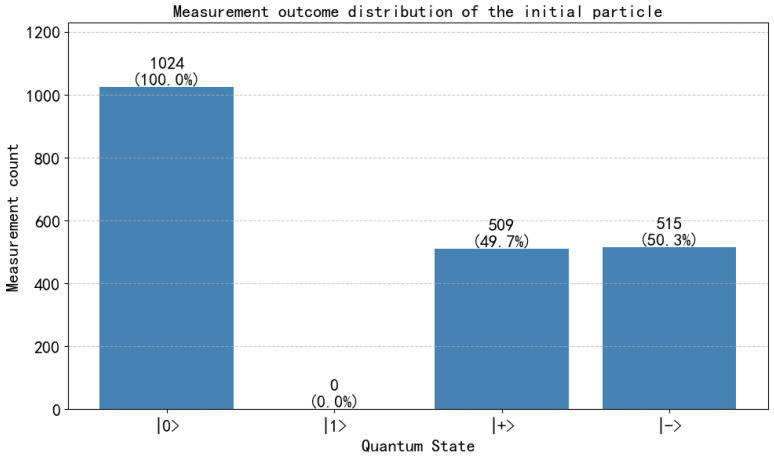
Grouping via the first rotation particle and unitary operations per group.

**Step 3:** HTP publishes (or broadcasts via classical channel) the measurement basis for the first rotation particle on the bulletin board while recording the number of bidders in each group. Bidders $P_{1},P_{2},P_{3}$ measure the first particle in their respective rotation particle sequences $Seq_{1}$, $Seq_{2}$ and $Seq_{3}$ using the announced basis. Taking $P_{1}$ as an example: when the first particle in its rotation sequence is ∣0〉 (Figure 6) and the computational basis measurement yields 0, $P_{1}$ is assigned to Group 1. Similarly, $P_{2}$ and $P_{3}$ are assigned to Group 2 and Group 3, respectively. These grouping results are consistent with the HTP’s recorded data. Then, Bidder $P_{1}$ performs unitary operations on positions 2–4 of its rotation particle sequence: specifically applying the $\sigma_{Y}$ operation to the 3rd position and the $\sigma_{I}$ operation to positions 2 and 4, while leaving other particles unchanged (Figure 7). Afterward, $P_{1}$ sends $Seq_{1}^{\prime }$ to HTP. Following the same rules, $P_{2}$ and $P_{3}$ execute their designated unitary operations and send their sequences $Seq_{2}^{\prime }$ and $Seq_{3}^{\prime }$ to HTP.

**Step 4:** Upon receiving the modified quantum sequences $Seq_{1}^{\prime }$, $Seq_{2}^{\prime }$ and $Seq_{3}^{\prime }$ from bidders $P_{1}$, $P_{2}$ and $P_{3}$, HTP performs sequential measurements on each qubit of the sequences using the measurement basis corresponding to each bidder’s group assignment. The mapping results are organized into a 3×12 matrix M, where each row represents a bidder’s sequence and each column corresponds to a specific qubit position.

**Figure 8:**
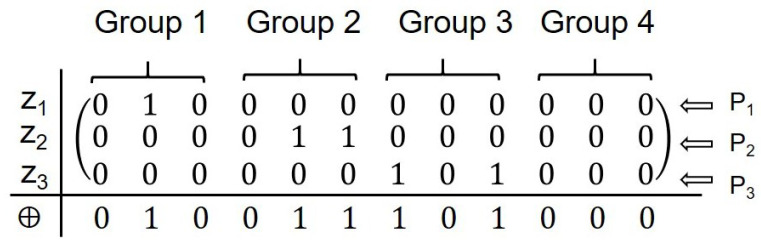
Mapping results arranged into a matrix for identity verification.

The per-column Hamming weights (column sums) are (0, 1, 0, 0, 1, 1, 1, 0, 1, 0, 0, 0). For every valid column, the sum equals 1; therefore, bidders $P_{1}$, $P_{2}$ and $P_{3}$ are assigned the identity designations Group 1 Member 2, Group 2 Member 3, and Group 3 Member 5, respectively, satisfying the predefined condition. Thus, the identity code distribution is successful without repetition (see Figure 8).

### 4.2. Multi-Party Semi-Quantum Simultaneous Ascending Auction

In this stage, all bidders simultaneously place bids on both auction items, A and B (though the protocol permits optional single-item bidding). For demonstration clarity, we assume single-round auction completion. Suppose the initial price ranges for items A and B in the first-round auction are [0, 8] and [0, 10], respectively, with a bid increment benchmark of 10% per round. In the first round, bidders $P_{1}$, $P_{2}$ and $P_{3}$ submit their initial bids for item A as (4, 7, 5) and for item B as (7, 7, 9). All bidders reveal their true maximum willingness-to-pay (i.e., their psychological reserve prices) in the first round, implying that no bidders will revise their bids in subsequent auction rounds.

**Step 1:** HTP initializes the protocol by assigning distinct key sequences $K_{1}^{\prime },K_{2}^{\prime },K_{3}^{\prime }$ to bidders $P_{1},P_{2},P_{3}$ respectively, where

$$\begin{array}{cc} K_{1}^{\prime } & =\{0,1,0,1,0,1,0,1,1,0,0,1,0,1,0,1,0,0,1,0,0,1,1,0,0\}\\ K_{2}^{\prime } & =\{1,0,0,1,1,1,0,1,0,0,0,0,1,0,1,0,1,1,0,0,0,0,1,0,1\}\\ K_{3}^{\prime } & =\{1,1,0,1,0,0,0,0,1,0,1,1,0,0,1,0,1,0,1,1,0,0,0,1,0\} \end{array}$$

Then, HTP randomly generates quantum sequences $Seq_{1}^{\prime \prime }$, $Seq_{2}^{\prime \prime }$ and $Seq_{3}^{\prime \prime }$ according to the rules of Step 1,where

$$\begin{array}{cc} {\mathrm{Seq}}_{1}^{\prime \prime } & =\{|0\rangle ,|-\rangle ,|+\rangle ,|0\rangle ,|1\rangle ,|+\rangle ,|0\rangle ,|0\rangle ,|+\rangle ,|-\rangle ,\\ & \;|+\rangle ,|0\rangle ,|+\rangle ,|1\rangle ,|-\rangle ,|-\rangle ,|+\rangle ,|0\rangle ,|1\rangle ,|+\rangle ,\\ & \;|-\rangle ,|+\rangle ,|0\rangle ,|1\rangle ,|+\rangle ,|+\rangle ,|0\rangle ,|+\rangle ,|1\rangle ,|-\rangle \}\\ {\mathrm{Seq}}_{2}^{\prime \prime } & =\{|-\rangle ,|1\rangle ,|-\rangle ,|-\rangle ,|+\rangle ,|+\rangle ,|+\rangle ,|1\rangle ,|+\rangle ,|1\rangle ,\\ & \;|0\rangle ,|+\rangle ,|-\rangle ,|-\rangle ,|+\rangle ,|0\rangle ,|+\rangle ,|1\rangle ,|-\rangle ,|-\rangle ,\\ & \;|+\rangle ,|0\rangle ,|+\rangle ,|1\rangle ,|-\rangle ,|0\rangle ,|+\rangle ,|1\rangle ,|-\rangle ,|-\rangle \}\\ {\mathrm{Seq}}_{3}^{\prime \prime } & =\{|-\rangle ,|0\rangle ,|+\rangle ,|+\rangle ,|1\rangle ,|-\rangle ,|0\rangle ,|+\rangle ,|-\rangle ,|1\rangle ,\\ & \;|-\rangle ,|0\rangle ,|+\rangle ,|1\rangle ,|-\rangle ,|+\rangle ,|1\rangle ,|-\rangle ,|-\rangle ,|+\rangle ,\\ & \;|0\rangle ,|+\rangle ,|1\rangle ,|-\rangle ,|-\rangle ,|+\rangle ,|-\rangle ,|-\rangle ,|+\rangle ,|0\rangle \} \end{array}$$

**Step 2:** Following the aforementioned rules, we conduct channel security eavesdropping detection by referencing the case study from Simulation Experiment 4.1.

**Step 3:** According to the established rules, bidders $P_{1}$, $P_{2}$ and $P_{3}$ perform group encoding operations on particles located at the first and second positions of the sequence, simultaneously executing identity encoding utilizing private vectors at positions 3 through 5.

As demonstrated in Simulation Experiment 4.1, the bidder $P_{1}$ was assigned the identity code “Group 1, Member 2” (with group encoding 00). Given $P_{1}$’s private vector (0, 1, 0), the identity encoding is implemented on qubits 1–5 through quantum operations: $P_{1}$ implements $\sigma_{I}$ operations on the first and second particles within their respective rotational particle; the $\sigma_{Y}$ operation is applied to the 4th qubit and $\sigma_{I}$ operations are maintained on qubits 3 and 5. Following the same rules, bidders $P_{2}$ and $P_{3}$ subsequently execute their respective unitary operations in accordance with their allocated group codes and private vectors.

**Step 4:**$P_{1}$, $P_{2}$ and $P_{3}$ convert the decimal bid value of item A into corresponding binary sequences (0, 1, 0, 0), (0, 1, 1, 1) and (0, 1, 0, 1). Concurrently, specific bid particle encoding is implemented at positions 6–9 to comprehensively embed auction information.

Taking bidder $P_{1}$ as an example, $P_{1}$ implement unitary operations on the rotational particle. Specifically, the $\sigma_{Y}$ operation is applied at positions 7, 11, 12, and 13, while the $\sigma_{I}$ operation is performed at positions 6, 8, 9, and 10. Then, $P_{1}$ sends $Seq_{1}^{\prime \prime \prime }$ to HTP. Following the same procedure, bidders $P_{2}$ and $P_{3}$ perform their corresponding quantum operations and return $Seq_{2}^{\prime \prime \prime }$ and $Seq_{3}^{\prime \prime \prime }$ to HTP.

**Figure 9:**
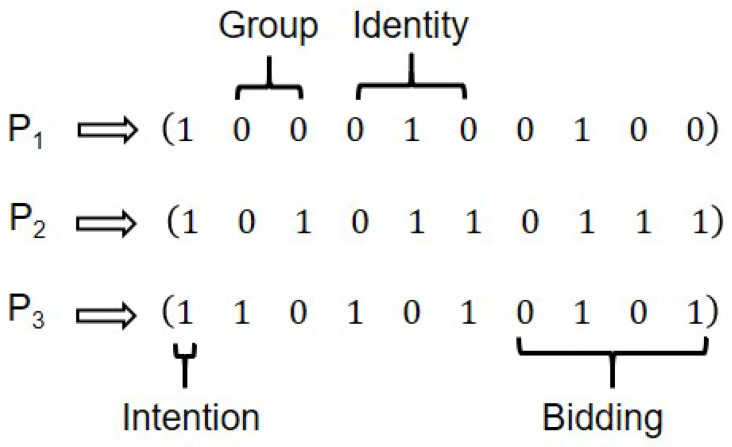
Recovered bid bits per item and round at HTP and highest-bid update.

**Step 5:** In accordance with the established rules, HTP constructs a 1×5 identity verification matrix (0, 0, 0, 1, 0), which matches the identity encoding assigned in Simulation Experiment 4.1. Subsequently, HTP conducts the following operations: (1) Using the same measurement basis as the initial state, HTP measures qubits 6–13 of $Seq_{1}^{\prime \prime \prime }$, obtaining a 1×8 matrix (0, 1, 0, 0, 0, 1, 1, 1) (Figure 9). The auction prices for items A and B are derived from their respective quantum sequences. When converted to decimal notation, these sequences yield the values 4 for A and 7 for B. The same verification procedure is applied to $Seq_{2}^{\prime \prime \prime }$ and $Seq_{3}^{\prime \prime \prime }$, revealing bids of (7, 7) and (5, 9) from $P_{2}$ and $P_{3}$, respectively, for item A and B.

(2) HTP announces the highest bid for item A in the first auction round as 7 on the public bulletin board. Item B yields respective bids of 7, 7 and 9 from $P_{1}$, $P_{2}$ and $P_{3}$, with the highest bid of 9 being similarly published. (3) Bidders $P_{1}$, $P_{2}$ and $P_{3}$ each generate hash commitments for their bids on items A and B according to Step 10. For example, for item A with bid 7, bidder $P_{1}$ samples a random nonce $s_{1}^{A}$ and computes which is recorded by HTP.

(3) $${\mathrm{com}}_{1}^{A}=\mathrm{SHA}-256(\mathrm{round}\_\mathrm{id}\;∥\;A\;∥\;{\mathrm{id}}_{1}\;∥\;7\;∥\;s_{1}^{A}),$$

**Step 6:** According to the auction rules, the starting prices for items A and B in the second round are set to 7.7 and 9.9, respectively, calculated as $\mathrm{Starting}\;{\mathrm{Price}}_{A-\mathrm{next}}=\left(1+10\%\right)\times 7=7.7$. Subsequently, HTP and bidders $P_{1}$, $P_{2}$ and $P_{3}$ repeat Steps 1–5 of the protocol. During Step 5, the bid matrices derived from the quantum sequences returned by the bidders to HTP are all (0, 0, 0, 0), indicating no new highest bids for either item. Next, the highest bidders for items A and B disclose their random numbers and commitment values to HTP and other bidders. Other bidders validate the commitment values, and HTP verifies both the commitment values and identity encodings.

If all verifications pass and no complaints are raised, HTP officially declares the highest bids for items A and B are 7 and 9, respectively. The winning bidders are Group 2 Member 3 for item A and Group 3 Member 5 for item B. The auction concludes upon this announcement.

## 5. Performance Analysis

We evaluate the proposed SQSAA protocol in terms of security, anonymity, verifiability, non-repudiation, and secrecy capacity.

### 5.1. External Attacks on Security

To obtain bidders’ secrets, an eavesdropper Eve may attempt intercept–resend, measure–resend, or Trojan-horse attacks.

#### 5.1.1. Intercept-Resend Attack

In Step 1 or Step 7, Eve may intercept the quantum sequence $Seq_{i}$ or $Seq_{i}^{\prime \prime }$ transmitted from HTP to $P_{i}$, insert an equivalent number of single particles, and subsequently forward the altered quantum sequence to $P_{i}$. Through this method, $P_{i}$ might encode the confidential information (identity codes or bidding information) onto these tampered particles. Eve then intercepts again the quantum sequence $Seq_{i}^{\prime }$ or $Seq_{i}^{\prime \prime \prime }$ sent from $P_{i}$ to HTP and obtains the identity codes or bidding information. It is noteworthy that the intercepted sequences $Seq_{i}$ or $Seq_{i}^{\prime \prime }$ contain decoy particles, with the probability of Eve’s fake particles matching the decoy particles being $\frac{1}{4}$. Without loss of generality, assuming the decoy particles prepared by HTP are in the ∣0〉 state, the detection probabilities for Eve’s fake particles are presented in Table 4.

Therefore, if the decoy particle is in the ∣+〉 state and HTP measures in the Fourier basis, the probability of detecting Eve is

(4) $$p=\frac{1}{4}\times 0+\frac{1}{4}\times 1+\frac{1}{4}\times \frac{1}{2}+\frac{1}{4}\times \frac{1}{2}=\frac{1}{2}.$$

Similarly, we can compute that the non-detection probabilities for ∣−〉, ∣0〉, and ∣1〉 are all $\frac{1}{2}$.

**Figure 10:**
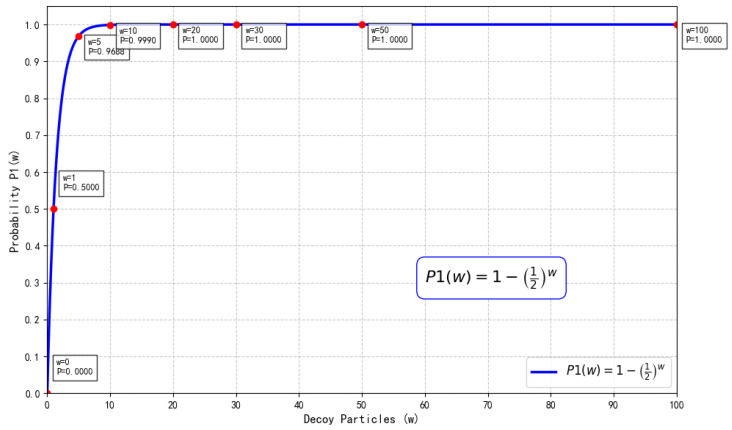
Detection probability $P_{1}\left(w\right)=1-2^{-w}$ versus number of decoy particles *w* under intercept-resend.

In summary, the probability that HTP detects Eve’s attack is given by

(5) $$P_{1}\left(w\right)=1-2^{-w},$$

where *w* represents the number of decoy particles (see Figure 10).

Consequently, Eve’s intercept-resend attack introduces an error rate in the eavesdropping detection phase that exceeds the threshold. Particularly when the number of decoy particles is sufficiently large, the probability of detecting the attack approaches 1, thereby effectively countering this attack.

#### 5.1.2. Measure-Resend Attack

In Step 1 or Step 7, Eve intercepts and measures the quantum sequences $Seq_{i}$ or $Seq_{i}^{\prime \prime }$ transmitted from HTP to $P_{i}$. Based on the measurement outcomes, she generates counterfeit quantum sequences $Seq_{i}$ or $Seq_{i}^{\prime \prime }$ and transmits them to $P_{i}$. Through this approach, $P_{i}$ may encode the secret information onto the tampered photons. Eve subsequently intercepts and measures the quantum sequences $Seq_{i}^{\prime }$ or $Seq_{i}^{\prime \prime \prime }$ sent from $P_{i}$ to HTP, thereby obtaining the secret information while simultaneously generating fake quantum sequences $Seq_{i}^{\prime }$ or $Seq_{i}^{\prime \prime \prime }$ to forward to HTP.

However, Eve possesses no prior knowledge regarding either the positions or preparation bases of the decoy particles. For each decoy particle, Eve has a statistical probability of 50% to select the correct measurement basis. If Eve chooses the correct measurement basis, she can successfully pass the eavesdropping detection. Conversely, if an incorrect basis is selected, Eve will be detected with a probability of $\frac{1}{2}$ statistically. Without loss of generality, assuming the decoy particles prepared by HTP are in the ∣+〉 state, the detection probabilities for Eve’s modified particles are presented in Table 5.

Similarly, we can compute that the detection probabilities for ∣−〉, ∣0〉, and ∣1〉 are all $\frac{1}{4}$. Therefore, if the decoy particle is in the ∣+〉 state, the probability of detecting Eve is

(6) $$p=\frac{1}{2}\times 1\times 0+\frac{1}{2}\times \frac{1}{2}\times 0+\frac{1}{2}\times \frac{1}{2}\times 1=\frac{1}{4}.$$

**Figure 11:**
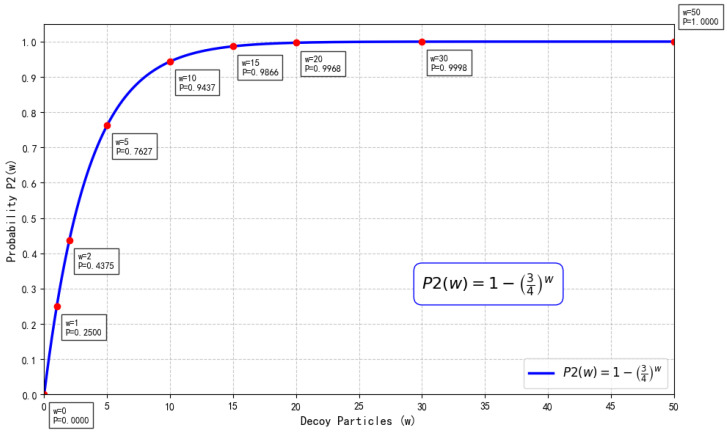
Detection probability $P_{2}\left(w\right)=1-{\left(3/4\right)}^{w}$ versus number of decoy particles *w* under measure-resend.

In summary, the probability that HTP detects Eve’s attack is given by

(7) $$P_{2}\left(w\right)=1-{\left(\frac{3}{4}\right)}^{w},$$

where *w* represents the number of decoy particles (see Figure 11).

In practical implementations, the sequence length *N* is finite. Asymptotic security bounds do not strictly apply. Statistical fluctuations in the estimated error rate must be considered. Let $n_{decoy}$ be the number of decoy bits. The probability that an attacker’s induced error deviates from the expected value by a margin δ is bounded by Hoeffding’s inequality. To guarantee a security failure probability $\epsilon_{sec}$, the threshold η must be adjusted (lowered) as the sequence length decreases, ensuring that any malicious intervention is statistically distinguishable from channel noise with high confidence.

#### 5.1.3. Trojan Horse Attack

Since the proposed protocol employs symmetric channels, it is vulnerable to Trojan horse attacks. A potential attacker, Eve, could exploit such attacks to intercept the private information of $P_{i}$. There are two primary types of Trojan horse attacks: delayed-photon attacks and invisible-photon attacks. To counter these attacks, each bidder $P_{i}$ needs to be equipped with both a wavelength quantum filter and a photon-number discriminator. The wavelength filter provides protection against invisible-photon attacks, while the photon-number splitter offers defense against delayed-photon attacks. These two devices have been demonstrated to exhibit strong resistance against Trojan horse attacks. Consequently, our protocol can effectively thwart Trojan horse attacks through the implementation of these devices.

### 5.2. Internal Attacks on Security

We focus on investigating an extreme case where n−1 dishonest users collaborate to attack the remaining single honest user. Without loss of generality, assume $P_{1}$ is the honest bidder while $P_{2},P_{3},\ldots ,P_{n}$ are dishonest bidders conspiring to attack $P_{1}$, with the objective of obtaining $P_{1}$’s secret information.

In the SQSAA protocol, since each $P_{i}$ and HTP communicate through star-shaped symmetric channels, the dishonest bidders would attempt to intercept particles transmitted between HTP and $P_{1}$ to acquire secret information. In this scenario, the dishonest bidders will be detected as external attackers. According to the protocol specifications, each bidder shares a private key with HTP, and the private key determines the positions of both decoy particles and rotation particles. Since dishonest bidders possess neither the private key shared between HTP and $P_{1}$ nor the information about quantum states prepared by HTP, any attack strategy they employ will inevitably cause quantum state disturbance and transmission errors and ultimately lead to an error rate exceeding the predetermined threshold. That is to say, our protocol can resist attacks from dishonest users.

### 5.3. Secrecy Capacity Analysis

**Figure 12:**
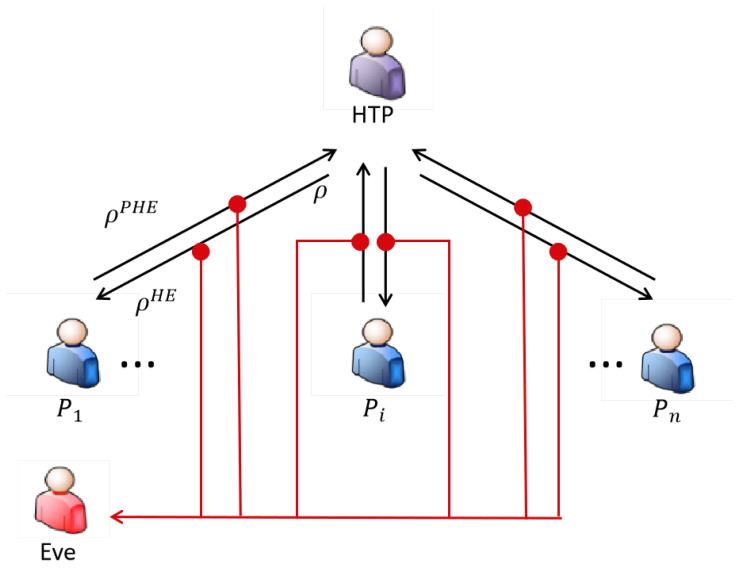
Wiretap channel model for secrecy capacity analysis in SQSAA.

Generally speaking, any secure communication protocol can be analyzed using the wiretap channel model, wherein the SQSAA protocol the quantum channel serving as the main channel transmits secret information while Eve’s eavesdropping is modeled as the wiretap channel, whose specific model is illustrated in Figure 12.

The secrecy capacity, serving as a fundamental metric for evaluating protocol security in quantum communications, represents the maximum secure information transmission rate between legitimate communicating parties (HTP and $P_{i}$) in the presence of eavesdropper Eve, which constitutes a critical parameter in SQSAA protocol analysis [35] and is expressed as $C_{S}$ according to Wyner’s wiretap channel theory.

(8) $$C_{s}=\max_{p}\left\{I\left(P_{i}:\mathrm{HTP}\right)-I\left(P_{i}:E\right)\right\}$$

where I(X:Y) characterizes the mutual information between random variables X and Y. Since the particles prepared by HTP have equal probabilities of being in $|0\rangle ,|1\rangle ,|+\rangle ,|-\rangle$ states, the system exhibits a completely mixed state $\rho =\frac{1}{2}\left(|0\rangle \;\langle 0|+|1\rangle \;\langle 1|\right)$. Under collective attack scenarios, Eve may perform joint operations on both the transmitted qubits and the ancillary qubits she prepares.

(9) $$\rho^{\left(HE\right)}=U\left(\rho ⊗|\epsilon \rangle \;\langle \epsilon |\right)U^{†}$$

where $|\epsilon \rangle$ denotes the state of Eve’s prepared ancillary qubit, and *U* is a unitary operator acting on the joint space of the qubit and ancillary qubit. Eve transmits the qubit to bidder $P_{i}$ while retaining her ancillary qubit until $P_{i}$ forwards the qubit to HTP. Upon reception, $P_{i}$ applies the $\sigma_{Y}$ operator with probability *p* or the $\sigma_{I}$ operator with probability (1 − *p*) based on secret information, evolving the qubit state to

(10) $$\begin{array}{cc} \rho^{\mathrm{PHE}} & =p\cdot \rho_{0}^{HE}+\left(1-p\right)\cdot \rho_{1}^{HE} \end{array}$$

where $\rho_{0}^{HE}=\sigma_{y}\rho^{HE}\sigma_{y}^{†}$ and $\rho_{1}^{HE}=\sigma_{I}\rho^{HE}\sigma_{I}^{†}$. To obtain the secret information, Eve performs coherent measurements on an arbitrary number of qubits and ancillary qubits to distinguish between the quantum states $\rho_{0}^{HE}$ and $\rho_{1}^{HE}$ encoded by bidder $P_{i}$, where based on the Holevo bound (whose distinguishing capability is bounded by the Holevo information quantity) we obtain

(11) $$\begin{array}{cc} I(P_{i}:E) & \leq \max\left\{S\left(\rho^{PHE}\right)-p\cdot S\left(\rho_{0}^{BE}\right)-\left(1-p\right)\cdot S\left(\rho_{1}^{BE}\right)\right\}\; \end{array}$$

where *S*(ρ) denotes the von Neumann entropy. The von Neumann entropy of the original quantum state $\rho =\frac{1}{2}\left(|0\rangle \;\langle 0|+|1\rangle \;\langle 1|\right)$ is $S\left(\rho \right)=-\left(\sum_{i=1}^{2}\frac{1}{2}\log_{2}\left(\frac{1}{2}\right)\right)=1$. Therefore, the maximum mutual information between bidder $P_{i}$ and eavesdropper Eve is

(12) $$\dot{I}\left(P_{i}:E\right)\leq \max\left\{S\left(\rho^{ABE}\right)\right\}-1\leq h\left(\xi \right),$$

where h(ξ) is the binary Shannon entropy of ξ.

(13) $$\begin{array}{cc} \xi & =\frac{1}{2}\left(1-\sqrt{{\left(1-2p\right)}^{2}+{\left[1-2\left(\epsilon_{X}+\epsilon_{Z}\right)\right]}^{2}\left[1-{\left(1-2p\right)}^{2}\right]}\right) \end{array}$$

where $\epsilon_{X}$ and $\epsilon_{Z}$ are the detection bit error rate (DBER) in the X-basis and the Z-basis, respectively. Taking into account channel losses, when denoting the maximum accessible qubit rate for Eve as $Q^{Eve}$, the maximum mutual information between bidder $P_{i}$ and Eve consequently becomes

(14) $$I\left(P_{i}:E\right)\leq Q^{\mathrm{Eve}}\cdot h\left(\xi \right)$$

Similarly, we denote the reception rate at HTP as $Q^{HTP}$ and calculate the mutual information between the bidder $P_{i}$ and HTP.

(15) $$I\left(P_{i}:HTP\right)=Q^{\mathrm{HTP}}\cdot \left[h(p+\epsilon -2p\epsilon )-h(\epsilon )\right]$$

Thus, the secrecy capacity $C_{S}$ becomes

(16) $$\begin{array}{cc} C_{S} & =\max\left\{I\left(P_{i}:\mathrm{HTP}\right)-I\left(P_{i}:E\right)\right\}\\ \; & =\max\left\{Q^{\mathrm{HTP}}\left[h(p+\epsilon -2p\epsilon )-h(\epsilon )\right]-Q^{\mathrm{Eve}}h\left(\xi \right)\right\}\\ \; & =Q^{\mathrm{HTP}}\cdot \max\left[h(p+\epsilon -2p\epsilon )-h(\epsilon )-g\cdot h(\xi )\right]\\ \; & \geq Q^{\mathrm{HTP}}\cdot \left[1-h\left(\epsilon \right)-g\cdot h\left(\epsilon_{X}+\epsilon_{Z}\right)\right] \end{array}$$

where *g* represents the discrepancy rate between $Q^{Eve}$ and $Q^{HTP}$, which depends on both channel loss and detector efficiency, reaching its maximum value at $p=\frac{1}{2}$.

### 5.4. Anonymity Analysis

According to the protocol, HTP transmits the quantum sequence $Seq_{i}$ to each bidder $P_{i}$ respectively, where the first particle of sequence $Seq_{j}$ received by the *j*-th bidder $P_{j}$ serves as the grouping particle. Since this particle is randomly prepared by HTP from $\left\{|0\rangle ,|1\rangle ,|+\rangle ,|-\rangle \right\}$ and the measurements of grouping particles among different bidders are mutually independent. Therefore, the mutual information between them can be quantitatively calculated as

(17) $$\begin{array}{cc} I(P_{j}:P_{l}) & =S\left(P_{j}\right)+S\left(P_{l}\right)-S\left(P_{j},P_{l}\right)\\ \; & =S\left(P_{j}\right)+S\left(P_{l}\right)-S\left(P_{j}\right)-S\left(P_{l}\right)\\ \; & =0. \end{array}$$

where $P_{l}$ denotes any dishonest bidder. Any collusion attempt by dishonest bidders to obtain $P_{j}$’s identity encoding is bound to fail. Attackers can neither access other bidders’ measurement results nor determine honest bidders’ group affiliation through the first particle, thus failing to reconstruct complete identity encoding. Furthermore, the decoy states introduced by HTP in $Seq_{i}$ ensure that any external eavesdropping will be detected due to induced errors during eavesdropping detection. Consequently, except for the trusted HTP, no bidder can obtain others’ identity information, guaranteeing the protocol’s anonymity.

The protocol guarantees that bidders cannot ascertain the identities or bids of other participants. However, it is important to note the Trusted HTP Assumption: The HTP knows the mapping between identity codes and real identities. Thus, the protocol provides anonymity against peers and outsiders, but not against the HTP.

### 5.5. Verifiability and Non-Repudiation Analysis

In Step 10 of the protocol, each bidder computes and submits the hash value of their bid to HTP, establishing a binding commitment since the original bid cannot be deduced without the random nonce. The protocol’s security guarantees non-repudiation, as any bid modification would require finding a new nonce matching the original hash value—a computationally infeasible task even for quantum computers due to the collision-resistant hash function.

Distinction from Channel Disruption: It is crucial to distinguish between *channel disruption* and *repudiation*. High QBER (Step 3) indicates an active attack or denial-of-service, leading to a protocol abort. In contrast, Non-Repudiation (Step 13) prevents a legitimate bidder—who has successfully transmitted a valid bid without triggering high QBER—from later denying their submission.

The verification phase (Step 13) requires the highest bidder to reveal their nonce and hash value for public verification, where successful verification leads to HTP disclosing the winner’s identity number and bid amount, while failed verification triggers a complaint mechanism that either initiates a new auction round for validated complaints or maintains the current highest bid for rejected complaints. This commitment verification framework satisfies the protocol’s verifiability requirement while maintaining quantum-resistant security throughout the auction process.

## 6. Conclusions

We presented a multi-party semi-quantum simultaneous ascending auction protocol based on single-particle states. HTP generates, distributes, and verifies quantum states; bidders determine groups via measurements and encode private identity vectors, obtaining identity codes after HTP authentication. During bidding, HTP dynamically updates sequences, and bidders submit prices for multiple items using only simple unitary operations. HTP announces current highest bids in real time and iteratively regenerates sequences until no higher bids appear, thereby realizing simultaneous multi-item ascending auctions.

The protocol serves as a quantum-secured signaling layer, providing information-theoretic security for bid transmission and identity verification over a classical auction mechanism. The protocol offers several practical advantages over prior art. First, it accommodates heterogeneous participants by requiring only semi-quantum capabilities on the bidder side (Z/X measurements, reflections, and elementary single-qubit operations), and it relies solely on single-particle resources, substantially lowering implementation cost. Second, it scales naturally to multi-item settings: identity is encoded in the first (2+n) particles, while each item uses $\left\lceil \log_{2}d_{w}\right\rceil$ particles for prices, enabling parallel, round-by-round price discovery. Third, the use of per-round hash commitments provides a simple, auditable path for public verification, supporting non-repudiation and complaint resolution without revealing secret randomness until the end.

From a deployment perspective, the protocol parameters are straightforward to tune. The decoy rate and QBER threshold η control eavesdropping detectability and robustness; the per-item bit width $\left\lceil \log_{2}d_{w}\right\rceil$ balances price granularity and sequence length; and the basic operation set (*I*, *H*, $\sigma_{Y}$, Z/X measurements) matches today’s photonic toolchains. Computational and storage complexity at bidders is minimal, and HTP’s classical processing (matrix checks and bit-to-decimal conversion) scales linearly in the number of bidders and items per round.

Finally, we must emphasize that theoretical validity does not automatically translate to immediate real-world applicability. Bridging the gap between our theoretical model and a fully operational real-world deployment remains a significant future challenge. This requires rigorous validation under realistic physical conditions, deep integration with existing technological infrastructures, and further study on practical device imperfections.

There remain limitations and opportunities for future work. The current model assumes a trusted HTP; relaxing this with distributed trust (e.g., threshold HTP, verifiable computation, or lightweight MPC among multiple centers) is an important direction. A finite-size and composable security analysis under realistic device imperfections and channel loss would strengthen the guarantees. On the systems side, building a prototype with time-bin or polarization-encoded photonics, integrating authenticated classical channels, and benchmarking end-to-end latency would support practical adoption. Finally, extending to richer auction formats (e.g., combinatorial or budget-constrained bidding), adaptive increment rules, and privacy-enhanced analytics while preserving semi-quantum feasibility are promising avenues.

## Figures and Tables

**Table 1:** Initial states and mapping results.

Initial States	Measurement Results	Mapping Results $z_{i}$
$|0\rangle$	$|0\rangle$	0
$|0\rangle$	$|1\rangle$	1
$|1\rangle$	$|1\rangle$	0
$|1\rangle$	$|0\rangle$	1
$|+\rangle$	$|+\rangle$	0
$|+\rangle$	$|-\rangle$	1
$|-\rangle$	$|-\rangle$	0
$|-\rangle$	$|+\rangle$	1

**Table 2:** Group mapping measurement and mapping results.

Initial State	Comp. Basis	Mapping	Fourier Basis	Mapping
$|0\rangle$	$|0\rangle$	0	$|+\rangle$	1
$|1\rangle$	$|1\rangle$	0	$|-\rangle$	1
$|+\rangle$	$|0\rangle$	1	$|+\rangle$	0
$|-\rangle$	$|1\rangle$	1	$|-\rangle$	0

**Table 3:** Example construction of sequences $Seq_{1},Seq_{2},Seq_{3}$ based on keys $K_{1},K_{2},K_{3}$ and BB84 states.

	1	2	3	4	5	6	7	8	9	10
$K_{1}$	0	1	0	1	0	1	0	0	1	0
$R_{1}$	∣0〉	∣1〉	∣+〉	∣1〉	∣1〉	∣0〉	∣0〉	∣0〉	∣−〉	∣−〉
$C_{1}$	∣0〉	∣−〉	∣−〉	∣0〉	∣+〉	∣+〉	∣−〉	∣0〉	∣+〉	∣−〉
$Seq_{1}$	∣0〉	∣−〉	∣+〉	∣0〉	∣1〉	∣+〉	∣0〉	∣0〉	∣+〉	∣−〉
$K_{2}$	1	0	0	0	1	1	0	1	0	0
$R_{2}$	∣+〉	∣1〉	∣−〉	∣0〉	∣0〉	∣−〉	∣+〉	∣1〉	∣+〉	∣1〉
$C_{2}$	∣−〉	∣+〉	∣1〉	∣−〉	∣+〉	∣+〉	∣−〉	∣1〉	∣1〉	∣0〉
$Seq_{2}$	∣−〉	∣1〉	∣−〉	∣0〉	∣+〉	∣+〉	∣+〉	∣1〉	∣+〉	∣1〉
$K_{3}$	1	1	0	1	0	0	0	0	1	0
$R_{3}$	∣−〉	∣−〉	∣+〉	∣0〉	∣1〉	∣−〉	∣0〉	∣+〉	∣−〉	∣1〉
$C_{3}$	∣−〉	∣0〉	∣1〉	∣+〉	∣−〉	∣−〉	∣0〉	∣0〉	∣−〉	∣+〉
$Seq_{3}$	∣−〉	∣0〉	∣+〉	∣+〉	∣1〉	∣−〉	∣0〉	∣+〉	∣−〉	∣1〉
	**11**	**12**	**13**	**14**	**15**	**16**	**17**	**18**	**19**	**20**
$K_{1}$	0	1	0	1	0	0	1	0	0	0
$R_{1}$	∣+〉	∣1〉	∣+〉	∣1〉	∣−〉	∣1〉	∣0〉	∣0〉	∣+〉	∣1〉
$C_{1}$	∣−〉	∣0〉	∣+〉	∣1〉	∣1〉	∣1〉	∣+〉	∣0〉	∣+〉	∣0〉
$Seq_{1}$	∣+〉	∣0〉	∣+〉	∣1〉	∣−〉	∣1〉	∣+〉	∣0〉	∣+〉	∣1〉
$K_{2}$	0	0	1	0	1	0	0	0	0	1
$R_{2}$	∣0〉	∣+〉	∣1〉	∣−〉	∣−〉	∣1〉	∣0〉	∣+〉	∣−〉	∣+〉
$C_{2}$	∣0〉	∣1〉	∣−〉	∣1〉	∣+〉	∣−〉	1	∣−〉	∣+〉	∣1〉
$Seq_{2}$	∣0〉	∣+〉	∣−〉	∣−〉	∣+〉	∣1〉	∣0〉	∣+〉	∣−〉	∣1〉
$K_{3}$	1	1	0	0	0	1	0	0	0	0
$R_{3}$	∣0〉	∣1〉	∣+〉	∣1〉	∣−〉	∣0〉	∣1〉	∣−〉	∣+〉	∣+〉
$C_{3}$	∣−〉	∣0〉	∣+〉	∣−〉	∣−〉	∣+〉	∣1〉	∣−〉	∣0〉	∣1〉
$Seq_{3}$	∣−〉	∣0〉	∣+〉	∣1〉	∣−〉	∣+〉	∣1〉	∣−〉	∣+〉	∣+〉

BB84 states include ∣0〉, ∣1〉, ∣+〉, and ∣−〉; *K* represents the key, *R* represents the receiver state, *C* represents the channel state, and Seq represents the resulting sequence.

**Table 4:** Measurement results and probabilities of fake particles.

Fake Particles	Measurement Result	Probability
∣+〉	∣+〉	0
∣−〉	∣−〉	1
∣0〉	∣+〉 or ∣−〉	1/2
∣1〉	∣+〉 or ∣−〉	1/2

The table shows the measurement outcomes and corresponding probabilities for different types of fake particles.

**Table 5:** Probabilities of Eve’s measurement choices and corresponding fake particles.

Eve’s Choice	Eve’s Measurement	Fake Particles	Probability
Fourier basis	∣+〉	∣+〉	0
Computational basis	∣+〉	∣+〉	0
Computational basis	∣−〉	∣−〉	1

“Eve’s Choice” refers to the basis selected by Eve for measurement; “Fake Particles” are the particles Eve replaces after measurement.

## Data Availability

The data presented in this study are available on request from the corresponding author.
